# Scrutinising the cytoskeleton

**DOI:** 10.1007/s00709-023-01857-3

**Published:** 2023-04-19

**Authors:** Peter Nick

**Affiliations:** Joseph Gottlieb Kölreuter Institute for Plant Sciences, Karlsruhe Institute of Technology, Karlsruhe, Germany

The discovery and manipulation of electromagnetic radiation with wavelengths smaller than those of visible light enabled, from the nineteen fourtees, imaging of physical objects down to atomar dimensions. However, the application of this strategy on biological objects was challenging because electrons have only a weak penetrance and, therefore, need to be handled in ultravacuum which means that even traces of water need to be avoided. Life without water is impossible, though. As a consequence, specimens have to be fixed while preserving their structure, then embedded, dehydrated, cut into ultrathin sections and contrasted by heavy metals (for review, see Pease and Porter 1981). Electron microscopy is, thus, the art of interpreting highly derived artifacts. Using this approach, filamentous structures became observable that spanned the cytoplasm as a seemingly architectural network, inspiring the term “cytoskeleton.” Originally, the search for this cytoskeleton was motivated by findings from outside of microscopy. For instance, searching for tubular structures that, based on biophysical arguments, had been predicted to guide the directional deposition of cellulose in plant cell walls (Green 1962) led Ledbetter and Porter (1963) to detect “microtubules,” while the nuclear lamina was discovered during the search for a mechanism sustaining nuclear shape (Pappas 1956). The very delicate actin filaments, in turn, could be visualised only many years after their biochemistry and function in motility had been studied in detail (Small et al. 1978). During the numerous decades following its discovery, the cytoskeleton turned out to be highly dynamic (at least for microtubules and actin), such that, with exception of the fairly static intermediate filaments, the term seems somewhat inappropriate from our current viewpoint. Despite this long history of research, many details of the cytoskeleton have remained elusive, especially with respect to functional diversification and evolution. Two contributions to the current issue scrutinise functional and evolutionary details of the cytoskeleton.

Deeper insight into the numerous functions of the cytoskeleton requires a correlation between changes of its organisation and the accompanying cellular response. This is far from trivial since the cytoskeleton appears as a complex and ever-changing three-dimensional structure that also differs to a certain extent between individual cells. Thus, a quantification of its morphological aspects is mandatory. The contribution by Yoshida et al. (2023) explores a machine-learning strategy to quantify the organisation of cortical microtubules in epidermal pavement cells of the model plant *Arabidopsis thaliana*. These cells provide mechanic constraints to the expanding leaves and, thus, decide about their size and shape. To withstand the forces exerted by the subtending turgescent cells, these pavement cells are interdigitated like the building blocks of a puzzle. Their complex shape depends on the organisation of the microtubular cytoskeleton. In fact, a mutant in a gene coding for a basic prolin-rich protein (bbp125), which binds to microtubules, exhibits a drastic change in cell shape, where the complex protrusions are missing, such that the pavement cells assume a more or less hexagonal shape without any interdigitation. Using this mutant as paradigm, the authors conduct a quantitative analysis of microtubule organisation (visualising them by means of a GFP-tagged tubulin) training an artificial intelligence focussing on the degree of microtubule alignment, their density and the coefficient of variation in the histogram as parameters. This strategy can assign a given cell with high accuracy either to a wild-type or a mutant condition, and allows the authors to define the contribution of individual parameters to prediction accuracy. They find that microtubule bundling is crucial in this respect and can demonstrate that density of microtubules increases while their alignment decreases in the bbp125 mutant. This is ascribed to changes in mechanical stress caused by the loss of the interdigitated cell shape. They further address the role of microtubule remodelling by application of taxol, an alkaloid, stabilising microtubules, and see here an increase in microtubule alignment linked with a partial loss of cell interdigitation, indicating that stable microtubules tend to align, a phenomenon which has been described previously for regenerating protoplasts that had been treated with taxol (Melan 1990). Thus, although the scope of this work is methodological, it also can serve as proof-of-concept that a more precise description of cytoskeletal organisation making use of novel strategies for quantitative image analysis can reveal mechanistic details that otherwise would have gone unnoticed.

In contrast to actin filaments and microtubules that are shared by the different eukaryotic clades, the evolution of intermediate filaments has remained more elusive. This is also reflected in a peculiar detail of lamins addressed by the contribution by Stick and Peter (2023). Lamins are imported into the nucleus and bind to the inner nuclear membrane by virtue of a CaaX motif recruiting an isoprenyl anchor, such that a network can form. This nuclear lamina sustains form and architecture of the nucleus in many eukaryotes, while missing from others, for instance the land plants and their algal ancestors Koreny and Field (2016). The progress in evolutionary genomics suggests that lamins were already present in the last eukaryotic common ancestor. Invertebrate animals, with a few exceptions, harbour only one lamin gene, while, in vertebrates, these genes are at least duplicated. On this background, the finding of invertebrates lacking lamins with a CaaX is astonishing since these species should not be able to produce a nuclear lamina. The three taxa belong to the so-called lophotrochozoan lineage. This group has been mainly defined by molecular evidence and replaced some of the classic taxonomic concepts, such as the Articulata, comprising earth worms and arthropods. The authors extend their analysis to seventeen phyla of this group and find that all Rotifera clades, but also taxa within the Mollusca and Annelida, lack the CaaX lamins, but instead harbour alternative C-termine rich in aromatic amino acids. Also, cases of differential splicing giving rise to variants with different C-termini are found. The functional interpretation of this curious difference is still open—while it is conceivable that also clusters of aromatic amino acids can confer membrane binding, this does not explain, why this happened in this particular lineage of the invertebrates. It might be a footprint of genetic drift during the time, when the first lophotrochozoan diverged. It might, however, indicate a second function in addition to the set-up of a nuclear lamina that counterselected against the isoprenyl anchor. This calls for an integration of cell biological traits with molecular phylogeny as recently demonstrated in an impressive manner for the evolution of the ciliary transition zone (Cavalier-Smith 2021).

More than 60 years after its discovery, the cytoskeleton has still retained many of its secrets. The contributions by Yoshida et al. (2023) and Stick and Peter (2023) show how new technology can complement classic cell biological analysis to unveil new and hitherto unknown facets. These new facets are inspiring curiosity in the first place. However, in order to lead to new knowledge, the new methodology needs to be integrated with functional analysis, thus transforming new data into new knowledge.
